# Data on biosurfactant assisted removal of TNT from contaminated soil

**DOI:** 10.1016/j.dib.2018.06.040

**Published:** 2018-06-22

**Authors:** Bijan Bina, Mohammad Mehdi Amin, Bahram Kamarehie, Ali Jafari, Mansour Ghaderpoori, Mohammad Amin Karami, Fahimeh Teimouri, Mohsen Sadani

**Affiliations:** a Environment Research Center, Research Institute for Primordial Prevention of Noncommunicable Disease, Isfahan University of Medical Sciences, Isfahan, Iran; b Dept. of Environmental Health Engineering, School of Health, Isfahan Univ. of Medical Sciences, Isfahan, Iran; c Department of Environmental Health Engineering, Faculty of Health and Nutrition, Lorestan University of Medical Sciences, Khorramabad, Iran; d Nutritional Health Research Center, Lorestan University of Medical Sciences, Khorramabad, Iran; e Environmental Science and Technology Research Center, Department of Environmental Health Engineering, Shahid Sadoughi University of Medical Sciences, Yazd, Iran; f Department of Environmental Health Engineering, School of Health, Shahid Beheshti University of Medical Sciences, Tehran, Iran

**Keywords:** TNT, Soil contamination, Rhamnolipid, Bioremediation, Soil

## Abstract

Contamination of environment, especially soil, is in great concern and can cause health problems. Thus, remediation of these pollutants through environmentally friendly methods should be considered. The aim of this data was bioremediation of TNT from contaminated soil. Two plastic pans were used as bioreactor. In each pan, 3 kg of soil was used. Concentration of TNT in contaminated soil was 1000 mg/kg. Rhamnolipid in concentration of 60 mg/l was added to intended pan. Sampling was done in each two weeks. In order to assessment of TNT degradation, samples were analyzed with HPLC. The data showed that after 154 days of experiment, TNT removal in soil that amended with rhamnolipid was 73% and in experiment with no addition of rhamnolipid was 58%. Based on the obtained data rhamnolipid was effective in remediation of TNT contaminated soil.

## Specifications Table

**Table:** 

Subject area	Biology
More specific subject area	Describe narrower subject area
Type of data	Table and figure
How data was acquired	High-performance liquid chromatography (HPLC) was used to track the TNT remediation. Degrading bacteria were identified with PCR technology.
Data format	Analyzed
Experimental factors	Assessment of TNT bioremediation in contaminated soil.
Experimental features	Soil was manually contaminated with TNT. In the 154 days of experiment duration, sampling was conducted every two weeks. For investigation of TNT degradation, the samples were analyzed with HPLC.
Data source location	Isfahan and Khorramabad, Iran
Data accessibility	Data are included in this article

## Value of the data

• The data may be useful for future researches that aimed in remediation of persistent organic pollutant.
• The data shows that aerobic remediation remove explosives (TNT) from contaminated soil.
• Aerobic remediation was effective in soil decontamination.
• The used approach can be useful for remediation of other explosives.

## 1 Data

**Fig. 1:**
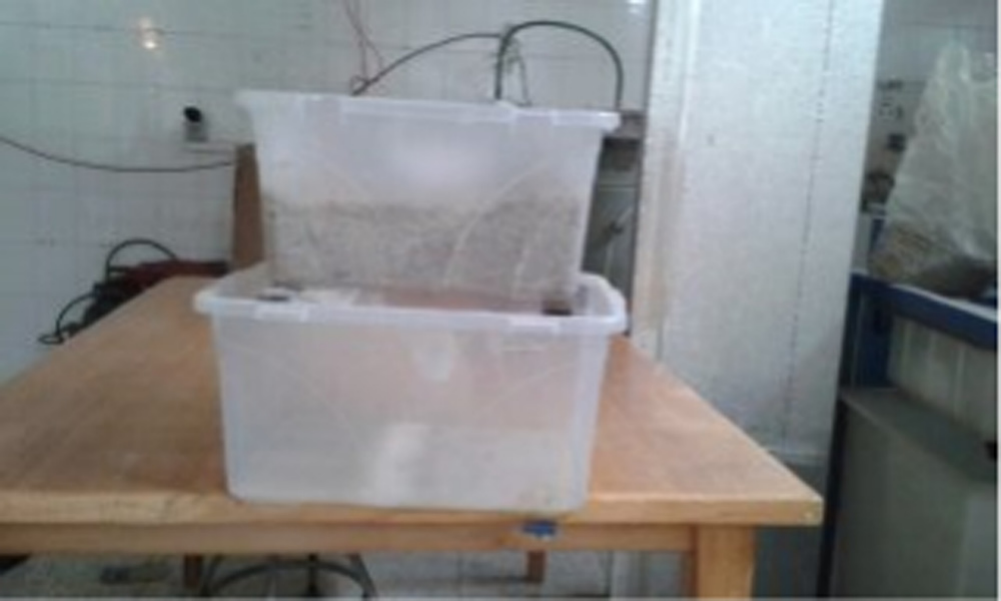
Schematic representation of the reactor used in this study.

**Fig. 2:**
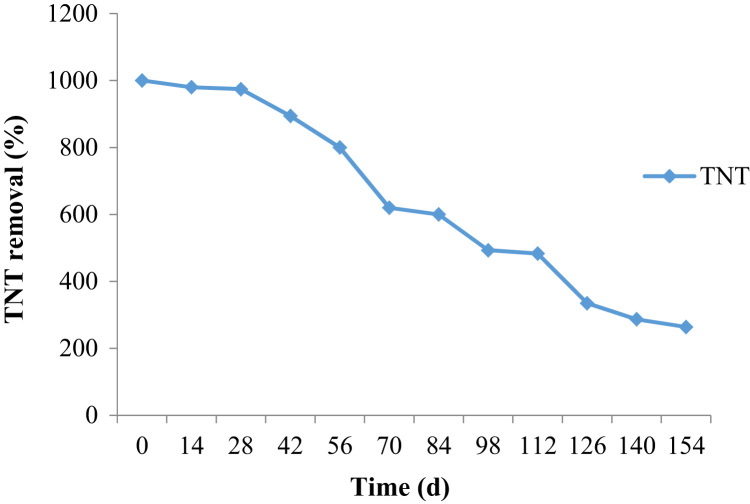
Removal of TNT from contaminated soil in rhamnolipid amendment experiment.

**Fig. 3:**
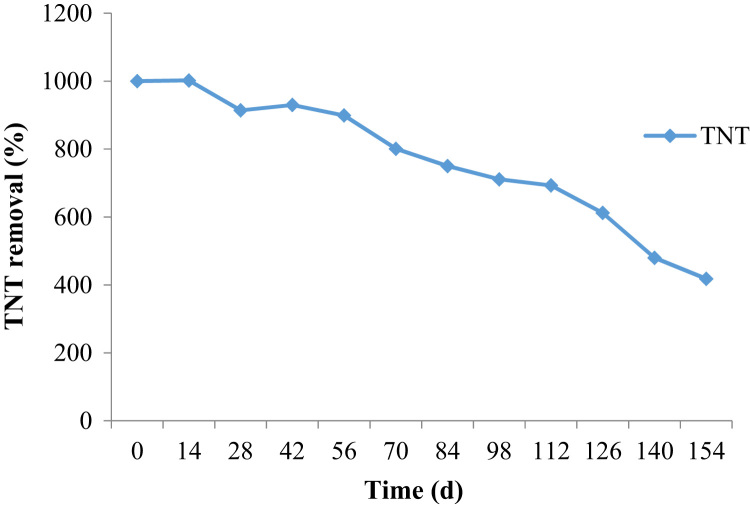
Removal of TNT from contaminated soil in the absence of rhamnolipid.

This brief dataset illustrates the natural remediation of TNT contaminated soil using aerobic bioremediation. The characteristic of contaminated soil is provided in Table 1. The used reactor is shown schematically in Fig. 1. The effect of parameters such as rhamnolipid addition and aeration on remediation of TNT is presented in Figs. 2 and Fig. 3. Identified degrading bacterial population is shown in Table 2. Bacterial population was shown in Table 3.

**Table 1:** Characteristic of contaminated soil.

**Parameter**	**Value (%)**
Clay	16
Sand	34
Silt	46
Total carbon	4

**Table 2:** Identified degrading bacteria.

**Experiment condition**	**Identified bacteria (Accession code)**
With rhamnolipid	Pseudomonas stutzeri (KF453954)
	Sphingomonadaceae (MF093198)

Without rhamnolipid	Rhodococcus (LN854587)

**Table 3:** Bacterial plate counts (colony forming units/g of soil).

**Experiment**	**30 (d)**	**60(d)**	**90 (d)**	**120 (d)**	**154 (d)**
Control	8 × 10^4^	17 × 10^4^	12 × 10^4^	15 × 10^4^	21 × 10^4^
Without rhamnolipid	4.8 × 10^6^	5 × 10^6^	6 × 10^6^	4.3 × 10^7^	7 × 10^6^
With rhamnolipid	1.4 × 10^4^	3 × 10^7^	4.8 × 10^7^	2.1 × 10^7^	2.2 × 10^7^

## 2 Experimental design, materials and methods

### 2.1 Soil preparation and TNT analyses

In this study, two soil bioreactors were used. Each bioreactor set consisted of a plastic pan (30 cm × 30 cm × 15 cm in height) that was placed in a slightly larger pan. In each pan, 3 kg of soil was used. The used soil was manually contaminated with TNT in a concentration of 1000 mg/kg. Contaminated soil was prepared by dissolving an appropriate amount of TNT in water/acetonitrile solution and a known weight of soil was then added with continuous mixing. The resultant mixture was placed in a ventilation hood to allow the complete evaporation of the solvent. The contaminated soil was then stored at room temperature for 7 days. The bottom of the smaller pan was perforated with 2-mm-diameter holes spaced 2 cm apart to allow the drainage of fluids during and after flooding phases. For controlling of moisture content, water was added every week. Aeration was done simultaneous with drainage by lifting of smaller pan vertically above the larger plastic pans. Due to low solubility of TNT [1], [2] rhamnolipid surfactant at concentration of 60 mg/l was added to contaminated soil in order to increasing its solubility. We assumed that rhamnolipid can led to the increase of TNT degradation. Rhamnolipid was added to the soil only at the beginning of the experiment. Simultaneous to the first irrigation, rhamnolipid at the above concentration was added to the contaminated soil. Applied dose was based on the critical micelle concentration (CMC). It is assumed that at CMC point, the pollutant was loaded in the micelles and subsequently its bioavailability increased. Rhamnolipid was purchased from National Institute for Genetic Engineering and Biotechnology, Institute of Chemistry and Chemical Engineering-Tehran, Iran. This study was conducted for 154 days. Sampling was done every two weeks. For assessing of TNT remediation, the gathered samples were analyzed according to the US EPA Method 8330 [1], [2], [3], [4], [5]. In this regard, 5 g (mixture of three grab samples) of soil was extracted with 20 ml of acetonitrile. The mixture was then filtrated with 0.22 μm Pall membrane. The prepared sample was analyzed with high-performance liquid chromatography (HPLC). The HPLC system used was from Waters (Milford, MA, USA), consisted of a Model 600E pump, Detector and a Nova-pak C18 The analytical column was an ODS2-Optimal column (25 cm × 4.6 mm id, 5 μm). A mixture of water-acetonitrile (20:80, v/v) was used as the mobile phase at a flow rate of 1.0 ml/min. The injection volume was 20 μl and the absorbance was measured at a wavelength of 210 nm.

### 2.2 Identification of isolates

In order to determine bacterial community composition, the PCR technology was used after 154 days of operation. After extraction of DNA from the soil, the universal eubacterial primers that consist of F27 (5′-AGAGTTTGATCMTGGCTCAG-3′) and R1492 (5′-TACGGYTACCTTGTTACGACT-3′), were used to amplifying the bacterial 16srDNA fragment (Table 2).
