# Supplementary material for: Data on biosurfactant assisted removal of TNT from contaminated soil
Source: Data Brief. 2018 Jun 22;19:1600–4. doi: 10.1016/j.dib.2018.06.040 (PMC6141866; doi:10.1016/j.dib.2018.06.040)
Supplement: Supplementary file 1 — Supplementary material [file mmc1.docx]

**Conflict of interest:**

The authors declare that there is no conflict of interest.
